# The 474-Kilobase-Pair Complete Genome Sequence of CeV-01B, a Virus Infecting *Haptolina* (*Chrysochromulina*) *ericina* (Prymnesiophyceae)

**DOI:** 10.1128/genomeA.01413-15

**Published:** 2015-12-03

**Authors:** Lucie Gallot-Lavallée, António Pagarete, Matthieu Legendre, Sebastien Santini, Ruth-Anne Sandaa, Heinz Himmelbauer, Hiroyuki Ogata, Gunnar Bratbak, Jean-Michel Claverie

**Affiliations:** a Structural and Genomic Information Laboratory (IGS), Aix-Marseille Université, CNRS UMR 7256 (IMM FR 3479), Marseille, France; b Department of Biology, University of Bergen, Bergen, Norway; c Institute of Biotechnology, University of Natural Resources and Life Sciences (BOKU), Vienna, Austria; d Institute for Chemical Research, Kyoto University, Uji, Japan; e Assistance Publique des Hôpitaux de Marseille (APHM), Marseille, France

## Abstract

We report the complete genome sequence of CeV-01B, a large double-stranded DNA virus infecting the unicellular marine phytoplankton *Haptolina* (formerly *Chrysochromulina*) *ericina.* CeV-01B and its closest relative *Phaeocystis globosa* virus define an emerging subclade of the Megaviridae family with smaller genomes and particles than the originally described giant Mimiviridae infecting *Acanthamoeba*.

## GENOME ANNOUNCEMENT

The haptophyte *Haptolina* (formerly *Chrysochromulina*) *ericina* is a phytoplankton species with a worldwide distribution. It most commonly occurs in low numbers but has occasionally been observed to form blooms (1). Viruses are abundant in aquatic ecosystems and are increasingly recognized to play a significant role in the regulation of plankton populations, such as in the prevention or termination of blooms (2). However, only a few different algal host-virus systems have been put in culture and studied in details. Comprehensive genome analyses have been performed for six DNA viruses infecting *Chlorella* spp. (3, 4), six infecting Mamiellales green algae (5–8), two infecting marine brown algae (Phaeophyceae) (9, 10), and one each infecting *Emiliania huxleyi* (Coccolithophyceae) (11), *Phaeocystis globosa* (Prymnesiophyceae) (12), and *Aureococcus anophagefferens* (Pelagophyceae) (13).

*Haptolina ericina* virus CeV-01B was isolated from Norwegian coastal waters in 1998 (1). The virus replicates in the host cytoplasm with a lytic cycle lasting 14 to 19 h resulting in thousands of icosahedral particles 160 nm in diameter. Its genome size was previously estimated around 510 kbp by pulsed-field gel electrophoresis (1). DNA from purified CeV-01B particles was sequenced in 2013 on an Illumina HiSeq 2000 Platform. 3,626,569 × 2 pair-ended 100-nt high-quality reads (approximately 1,400-fold coverage of the CeV-01B genome) were generated and assembled using SOAPdenovo (14) with a stringent k-mer size (*k* = 97). Two scaffolds, with sizes of 67 kb and 410 kb were initially obtained. The scaffolder SSPACE (15), Gapfiller (16), and Bowtie (17) were used to fill up the gaps and to correct remaining sequencing errors.

The final 473,558-bp genome sequence exhibited a high A+T content of 75%. It was predicted to encode 512 open reading frames (ORFs), using GeneMark (18), and 12 tRNAs, using tRNAscan-SE (19). They span over 91% of the genome. Among the 512 predicted ORFs, 274 (53.5%) exhibited a significant homolog in NCBI’s nonredundant protein sequence database (BlastP, *E* value < 10^−5^), of which 163 (59.5%) were most similar to their PgV homolog and an additional 40 had their closest homologue in other Megaviridae. These best matches in PgV include usual phylogenetic markers such as the DNA polymerase B (CeV_365, 45% identical to PgV’s PGCG_248), the major capsid protein (CeV_191, 73% identical to PgV’s PGCG_157), as well as two enzymes characteristic of Megaviridae: the mismatch DNA repair enzyme MutS7 (20) (CeV_281 47% identical to PgV’s PGCG_223) and an asparagine synthetase (21) (CeV_376, 49% identical to PgV’s PGCG_327). Moreover, CeV and PgV share gene fusion between their DNA polymerase X and NAD-dependent DNA ligase (CeV_489, 49% identical to PGCG_401) not found in other Megaviridae. Finally, the newly described PgV-MIGE mobile element of which 12 copies were found in the PgV genome (13) was also found in 6 copies in the CeV-01B genome.

The proposed Megaviridae family, initially composed of *Acanthamoeba*-infecting giant viruses (now constituting the proposed Mimiviridae subfamily) (21), progressively expanded to encompass smaller members infecting other unicellular protists (12, 13, 21). Its genome sequence clearly classifies CeV-01B in a subclade of “small” Megaviridae (12) with PgV as the closest, nevertheless distant relative.

### Nucleotide sequence accession number.

The completely annotated genomic sequence of *Haptolina ericina* virus CeV-01B is available in Genbank under accession number KT820662.
